# Novel *TNIP2* and *TRAF2* Variants Are Implicated in the Pathogenesis of Pulmonary Arterial Hypertension

**DOI:** 10.3389/fmed.2021.625763

**Published:** 2021-04-30

**Authors:** Shaun Pienkos, Natalia Gallego, David F. Condon, Alejandro Cruz-Utrilla, Nuria Ochoa, Julián Nevado, Pedro Arias, Stuti Agarwal, Hiral Patel, Ananya Chakraborty, Pablo Lapunzina, Pilar Escribano, Jair Tenorio-Castaño, Vinicio A. de Jesús Pérez

**Affiliations:** ^1^Division of Pulmonary and Critical Care Medicine and Department of Medicine, Stanford University, Stanford, CA, United States; ^2^Medical and Molecular Genetics Institute (INGEMM), IdiPaz, Hospital Universitario La Paz, Madrid, Spain; ^3^CIBERER, Centro de Investigación en Red de Enfermedades Raras, Instituto de Salud Carlos III, Madrid, Spain; ^4^Pulmonary Hypertension Unit, Department of Cardiology, Hospital Universitario Doce de Octubre, Madrid, Spain; ^5^Centro de Investigación Biomedica en Red en Enfermedades Cardiovasculares, Instituto de Salud Carlos III (CIBERCV), Madrid, Spain; ^6^Intellectual Disability, TeleHealth, Autism and Congenital Anomalies (ITHACA), European Reference Network on Rare Congenital Malformations and Rare Intellectual Disability, Brussels, Belgium

**Keywords:** pulmonary arterial hypertension, NF-κB, inflammation, massive paralleled sequencing, *TNIP2*, *TRAF2*

## Abstract

**Background:** Pulmonary arterial hypertension (PAH) is a rare disease characterized by pulmonary vascular remodeling and right heart failure. Specific genetic variants increase the incidence of PAH in carriers with a family history of PAH, those who suffer from certain medical conditions, and even those with no apparent risk factors. Inflammation and immune dysregulation are related to vascular remodeling in PAH, but whether genetic susceptibility modifies the PAH immune response is unclear. *TNIP2* and *TRAF2* encode for immunomodulatory proteins that regulate NF-κB activation, a transcription factor complex associated with inflammation and vascular remodeling in PAH.

**Methods:** Two unrelated families with PAH cases underwent whole-exome sequencing (WES). A custom pipeline for variant prioritization was carried out to obtain candidate variants. To determine the impact of TNIP2 and TRAF2 in cell proliferation, we performed an MTS [3-(4,5-dimethylthiazol-2-yl)-5-(3-carboxymethoxyphenyl)-2-(4-sulfophenyl)-2H-tetrazolium] assay on healthy lung pericytes transfected with siRNA specific for each gene. To measure the effect of loss of TNIP2 and TRAF2 on NF-kappa-beta (NF-κB) activity, we measured levels of Phospho-p65-NF-κB in siRNA-transfected pericytes using western immunoblotting.

**Results:** We discovered a novel missense variant in the *TNIP2* gene in two affected individuals from the same family. The two patients had a complex form of PAH with interatrial communication and scleroderma. In the second family, WES of the proband with PAH and primary biliary cirrhosis revealed a *de novo* protein-truncating variant in the *TRAF2*. The knockdown of TNIP2 and TRAF2 increased NF-κB activity in healthy lung pericytes, which correlated with a significant increase in proliferation over 24 h.

**Conclusions:** We have identified two rare novel variants in *TNIP2* and *TRAF2* using WES. We speculate that loss of function in these genes promotes pulmonary vascular remodeling by allowing overactivation of the NF-κB signaling activity. Our findings support a role for WES in helping identify novel genetic variants associated with dysfunctional immune response in PAH.

## Introduction

Pulmonary arterial hypertension (PAH) is a rare disorder associated with progressive elevation of pulmonary pressures that, if untreated, leads to right heart failure and death (1, 2). The major pathological features of PAH are obliterative vasculopathy and progressive loss of distal pulmonary microvessels that are unresponsive to available therapies (3, 4). While the cause of PAH remains incompletely understood, pathogenic variants in the bone morphogenetic protein receptor 2 *(BMPR2)* have been associated with both familial (60%) and sporadic PAH (20–25%) (5, 6). Studies have now shown that BMPR2 is responsible for ensuring appropriate vascular repair in response to injury and loss of function variants are associated with impaired angiogenesis, as well as endothelial and smooth muscle cell proliferation within the vessel wall (7–9). Given the low penetrance of *BMPR2* variants, it is likely that additional genetic modifiers are required for the development of PAH. Besides genetic predisposition, immune dysregulation and inflammation have been established as risk factors for PAH development (10, 11). Recent work in this area has led to identifying immune subtypes within PAH, which may explain phenotypic differences among patients (12). Understanding the interaction between genetics and immunity could provide an avenue for revealing the basis of PAH susceptibility and might offer opportunities for novel therapeutics that could change the natural history of this disease.

Whole-exome sequencing (WES) is a valuable tool for discovering novel pathogenic variants in the protein-coding regions and the genome's intron-exon boundaries. The use of WES to analyze probands and family members (i.e., trios and quartets) has led to the identification of novel disease genes associated with multiple genetic disorders, including PAH (13–16). In this study, we employed WES to detect novel pathogenic variants in a patient trio and a quartet that had previously tested negative for known PAH pathogenic variants using our customized parallel sequencing PAH gene platform (17). We report the discovery of potentially pathogenic variants in *TNIP2* and *TRAF2*, two genes associated with inflammation and immunity whose role in PAH has not been previously documented. Through the use of patient-derived cells and *in vitro* gene knockdown studies, we show that TNIP2 and TRAF2 act by suppressing the activation of NF-κB, a transcription complex that controls the expression of inflammatory cytokines and genes associated with cell proliferation in PAH (18–21). We conclude that *TNIP2* and *TRAF2* pathogenic variants could increase PAH susceptibility through their capacity to alter cellular immune responses and drive abnormal cellular proliferation in the pulmonary vasculature.

## Materials and Methods

### Patient Studies

All patients involved in this study gave their informed consent to participate. Samples were obtained from the Spanish PAH registries (REHAP and REHIPED), and the ethical committee approved the project of scientific research of the Hospital Universitario La Paz, Madrid (PI-1210, PI-3996). PAH was confirmed in all patients by right heart catheterization (RHC) following the recommendations of the 6th World Symposium and the European Society of Cardiology (1, 2, 22). Screening of family members was carried out by performing a family pedigree of all individual index cases of PAH. In those with a clinical suspicion of heritable PAH, evaluation of medical history, physical examination, laboratory tests, 12-lead electrocardiogram, and transthoracic echocardiography of all family members was done to select possible affected members and RHC candidates.

### Whole Exome Sequencing

The workflow used for WES analysis is summarized in Supplementary Table 1. Library preparation was carried by Agilent SureSelect^TM^ (v. 6.0), all exons kit followed by sequencing in a NovaSeq Sequencer (Illumina, USA). The exomes were analyzed by Saphetor Varsome software (23) and VarSeq (Golden Helix, USA) to prioritize variants in the probands with segregation patterns consistent with potential disease pathogenesis. Copy number variant (CNV) analysis was carried out through VarSeq to detect large rearrangements (deletions, insertions, and Indels of more than 1 Kb). Segregation analysis and validation of the variants detected in the WES was performed by classical Sanger sequencing. Screening for pathogenic variants in well-known PAH genes was carried out through a custom gene panel described previously (17).

Variant prioritization was carried out by a custom algorithm (see Supplementary Table 1). The first step was to filter out variants not meeting the standard quality requirements for depth, genotype quality, and variant allele frequency, followed by segregation analysis based on the variant's suggested pattern of inheritance. All variants with a population frequency above 1% were filtered out, and the pathogenicity of the remaining variants was assessed using several bioinformatic tools included in the dbNSFP database (24) plus the computation of the CADD score, which predict the deleteriousness of variants throughout the human genome (25). The variant analysis was prioritized according to predicted pathogenicity. Predicted impact of variants on candidate gene function and role in PAH pathogenesis was carried out using *in silico* analysis via STRING and literature review, respectively.

### Pericyte Isolation and Culture

Pericytes were obtained from human donor lung tissue samples and lineage was confirmed by FACS, and western immunoblotting as previously described (26–28). 3G5 (ATCC CRL-1814) hybridoma cells were cultured in 1X DMEM with 10% FBS. Culture medium was collected and concentrated by centrifugal filter Amicon Ultra-15 (Millipore). 3G5 IgM was isolated using the IgM Purification Kit (Pierce), and concentration was determined using the Lowry Method. The day before the extraction, 40 μl of rat anti-mouse IgM magnetic beads (Invitrogen 11039D) were incubated with 10 μg of 3G5 IgM overnight. The next day, fresh lung tissue was washed with 1X HBSS twice and digested in a 10 ml 1X HBSS solution containing 10 mg Collagenase (Sigma-Aldrich, USA), 1 mg dispase, and 75 μg DNase for 15–30 min at 37 degrees on an orbital shaker at vigorous speed (150 rpm). An equivalent amount of ice-cold pericyte medium (ScienCell, USA) containing 2% FBS was added immediately after digestion. The suspension was passed through a 100 μm mesh filter (BD Falcon, USA) to remove debris and undigested fibrous tissue and washed once with 1X PBS. The cell pellet was resuspended in 1 ml 1X PBS containing 0.2% FBS and 40 μl of antibody-coated magnetic beads and gently rotated in 4 degrees for 45 min. Cells were used between Passage 8–10.

### Western Immunoblotting

Cells were washed three times with ice-cold 1x PBS, and lysates were prepared by adding lysis buffer (1X radio-immunoprecipitation assay buffer, 1 mMol/L phenyl-methylsulfonyl fluoride, and 1x phosphatase inhibitor) and vortexed for 10 s before centrifugation. Supernatants were transferred to fresh microcentrifuge tubes and stored at −20°C. The protein concentration was determined by Pierce BCA assay (Thermo Scientific Product #23227, Rockford, Illinois). Equal amounts of protein were loaded onto each lane of a 4–12% Bis-Tris gel (Life Technologies) and subjected to SDS-PAGE electrophoresis under reducing conditions. After blotting, polyvinylidene difluoride (PVDF) membranes were blocked for 1 h in blocking buffer (5% milk powder in 0.1% PBS/Tween 20) and incubated with primary antibodies overnight at 4°C, followed the next day by incubation with horseradish peroxidase-conjugated secondary antibodies. Bands were visualized using the ECL kit (Thermo Fisher).

### RNA Interference

To achieve gene knockdown, 2 μmol/L silencing RNA (siRNA) of ABIN2 (encoded by TNIP2) (L-014328-01, Dharmacon, Lafayette, CO), TRAF2 (L-005198-00, Dharmacon, Lafayette, CO), or non-targeting siRNA control transfected into healthy pulmonary pericytes. Transfection was performed using a Nucleofector II (Program U-025; Lonza) with the Basic Smooth Muscle Cell Nucleofection kit (Lonza). All experiments were performed 48 h after electroporation.

### MTS Proliferation Assay

Cells were seeded in eight replicates on collagen-coated 96-well plates (5.0 × 10^3^ cells per well) (Nunc, Rochester, NY) and cultured in complete medium overnight at 37°C and 5% CO_2_. The next day, reduced serum media and MTS reagent were added to each well for 1 h in accordance with the manufacturer's instructions. MTS absorbance was measured at 490 nm.

### Statistical Analysis

The number of samples studied per experiment is indicated in the figure legends. Values from multiple experiments are expressed as mean +/– SEM. Statistical significance was determined using the Mann-Whitney test. A value of *P* < 0.05 was considered significant.

## Results

### Discovery of the *TNIP2* Variant

**Figure 1:**
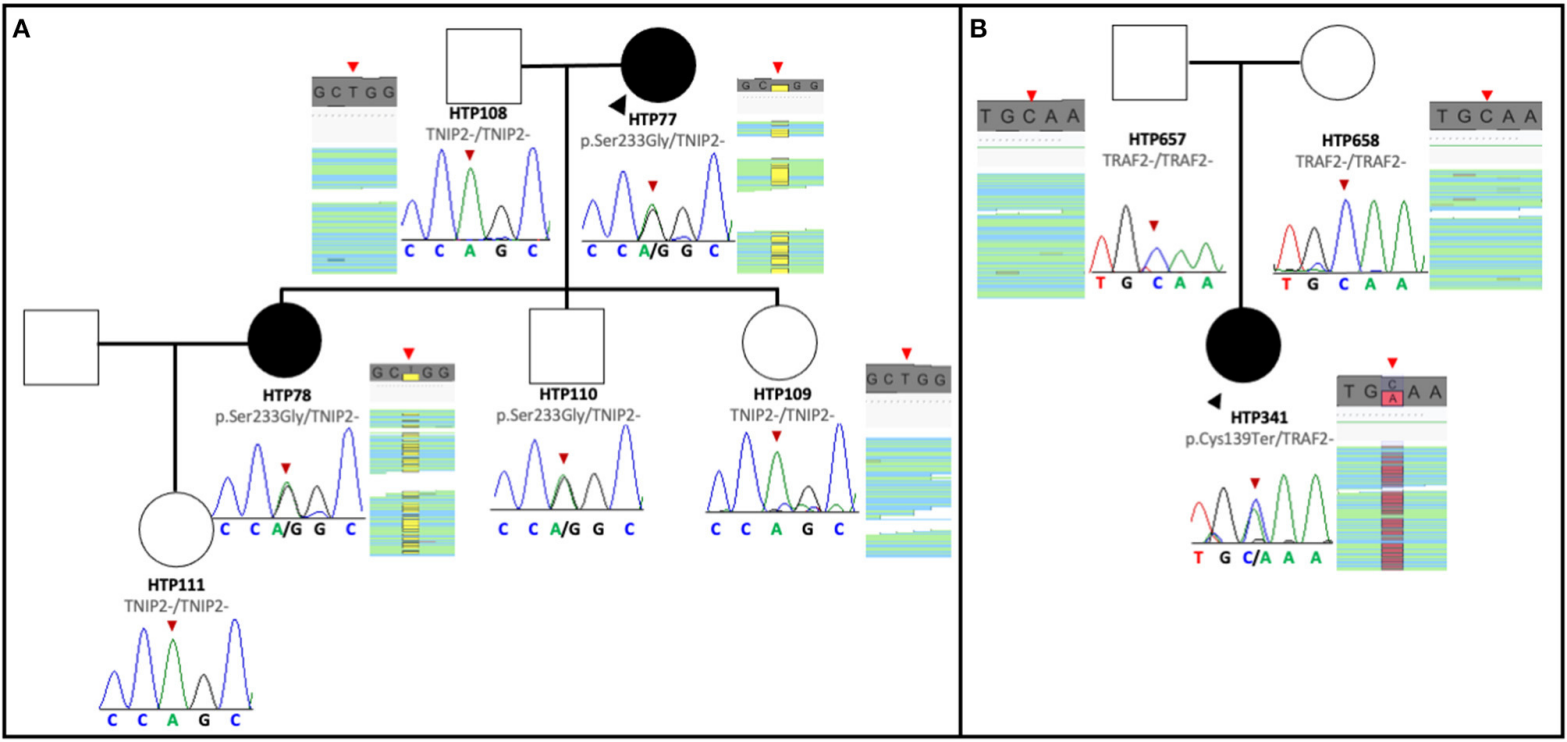
Pedigree of the families with *TNIP2* and *TRAF2* variants. **(A)** The *TNIP2* variant was found in two affected family members and an unaffected sibling of the proband. Sanger sequencing segregation in all available members is presented, with BAM capture from the IGV software showing the vertical read coverage at the specific position (red arrows). **(B)** Pedigree of the family with the *TRAF2* variant. The nonsense variant was detected only in the proband and confirmed with Sanger sequencing of the variant. BAM capture from the IGV software showing the vertical read coverage at the specific position (red arrows).

The pedigree of the first family is shown in Figure 1A. The index case was a 63-year-old female with a history of Ostium Secundum atrial septal defect (ASD), which was surgically corrected in 1994, and severe restrictive lung disease caused by thoracic deformities and diaphragmatic paralysis. The patient was diagnosed with PAH when she presented with exertional dyspnea and chest pain. Despite treatment with sildenafil and ambrisentan, the patient died 4 years after diagnosis from progressive right heart failure. Her 45-year-old daughter was diagnosed in 2015 with PAH associated with limited scleroderma and remains clinically stable with triple therapy of tadalafil, bosentan, and intravenous epoprostenol. The clinical characteristics of the two affected patients are summarized in Table 1. The remaining family members were screened and demonstrated no signs and symptoms of PAH to date. Given the familial predisposition, we conducted a genetic screen of the patients and family members using an in-house custom panel (HAPv2.1) (17), which was negative for mutations in 21 genes associated with PAH. As a next step, we conducted WES on the two patients and four unaffected family members, followed by segregation analysis (Figure 1A).

**Table 1:** Clinical features of patients.

**Patient ID**	**HTP 77**	**HTP 78**	**HTP 341**
Etiology	Group 1: PAH + ASD	Group 1: PAH + scleroderma	Group 1: PAH + primary biliary cirrhosis
Date of birth	22/09/1946	15/10/1975	24/08/1988
Age at diagnosis (years)	63	27	27
Current age (years)	Dead with 67 years	45	32
WHO functional class	III	I	I
Last known PAH therapies	Sildenafil, ambrisentan	Bosentan, tadalafil, epoprostenol	Selexipag, macitentan, tadalafil
6-min walk test (meters)	Not performed	595	454
CPET (date) RER Maximum V02—mL/Kg/min (% of predicted value) VE/VCO2 slope	Not performed	May of 2018 1.41 11.0 (33) 33	March of 2018 1.15 17.0 (53) 38
Mean pulmonary artery pressure (mmHg)	42	51	55
Pulmonary capillary wedge pressure (mmHg)	14	6	8
Pulmonary vascular resistance (Wood units)	6.0	9.1	17.0
Cardiac output (lpm)	4.6 lpm	4.6 lpm	2.7 lpm
Cardiac index (lpm/m^2^)	3.0	3.1	1.8
Mean arterial pressure or blood pressure (mmHg)	96	83	74
Echocardiographic abnormalities	Repaired Ostium Secundum ASD. Mild dilatation and hypertrophy of the RV and mild RV systolic dysfunction. Mild RA enlargement.	Eccentricity index of 1.7. RV hypertrophy and dilatation. RV systolic dysfunction. Moderate RA enlargement.	Eccentricity index of 1.1. RV hypertrophy and dilatation. Subtle RV systolic dysfunction. Mild RA enlargement.
Chest computed tomography abnormalities	Thoracic deformities and diaphragmatic paralysis	Bilateral ground glass opacities related with PH.	No abnormalities noted in chest CT Arterio-venous hepatic fistulae
Forced expiratory volume in 1 s (mL) and predicted value by age and sex (%)	660 (43%)	2,790 (86%)	2,00 (89%)
Forced vital capacity (mL) and predicted value by age and sex (%)	1,060 (56%)	3,240 (61%)	3,185 (91%)
Diffusing capacity of the lungs for carbon monoxide (DLCO)	Not available	45%	56%
Partial pressure of oxygen (PaO_2_)	75 mmHg	57 mmHg	55 mmHg
V/Q scintigraphy and/or CT pulmonary angiography	Global matched perfusion and ventilation defects	Normal V/Q scan. Main pulmonary artery enlargement. RA and RV enlargement	Matched perfusion and ventilation defects in the upper right lung segment in V/Q scan.

*Summary of the most relevant clinical characteristics of the patients in which a genetic variant was detected. ASD, atrial septal defect; CPET, cardiopulmonary exercise test; CT, Computed tomography; PH, pulmonary hypertension; RA, right atrium; RER, respiratory exchange ratio; RV, right ventricle; V/Q, Ventilation-Perfusion scan; WHO, World Health Organization*.

Analysis of WES data confirmed the absence of mutations in PAH associated genes. However, we discovered that both PAH patients were carriers of a novel missense variant in TNFAIP3 Interacting Protein 2 (*TNIP2*). This gene encodes for the protein ABIN2, which acts as an inhibitor of the NF-κB signaling pathway (29). The variant is predicted to change an adenine to a cytosine base at position 697 (NM_024309.4:c.697A>C), which results in a switch from serine to glycine at position 233 [p.(Ser233Gly)] of the peptide chain, located between the two ubiquitin-binding domains of the protein. This novel *TNIP2* variant was predicted to be damaging based on the estimates obtained via dbSNFP and would likely result in loss of protein function (Table 2). Additionally, this variant is absent from several control population databases (gnomAD exomes, gnomAD genomes, Kaviar, Beacon, Bravo, ESP, 1000G phase III). Interestingly, one of the unaffected family members was also found to be a carrier of the same *TNIP2* variant and is currently undergoing clinical surveillance in our clinic.

**Table 2:** Candidate genes and variant *in silico* analysis.

**Variant information (hg19)**					**Pathogenicity**		**Population frequencies**
**Gene**	**cDNA position**	**Protein position**	**Effect**	**Zygosity**	**dbSNFP**	**CADD**	**AF**
*TNIP2*	NM_024309.3:c.697A>G	NP_077285.3:p.Ser233Gly	Missense	Heterozygous	12/19	25.8	0
*TRAF2*	NM_021138.3:c.417C>A	NP_066961.2:p.Cys139Ter	Non-sense	Heterozygous	5/9	35	0

*Variants were annotated with the human reference genome hg19. Pathogenicity in silico prediction was obtain from the aggregation database dbSNFP and CADD (Table 1 for more info). AF, Alleled frequency was estimated from several population pseudo-control databases: gnomAD genomes (v2.1.1), gnomAD exomes (v2.1.1), Kaviar (version 160204-Public), Beacon (v2.0), 1000G, Phase III and Bravo*.

### Discovery of the *TRAF2* Variant

The pedigree of the second family is shown in Figure 1B. The index case was a 27-year-old female that presented to the clinic in 2015 complaining of dizziness and was subsequently diagnosed with idiopathic PAH. Following initiation of tadalafil and ambrisentan, the patient developed elevated liver function enzymes, which prompted ambrisentan cessation. During the workup for elevation in liver enzymes, the patient was diagnosed with primary biliary cirrhosis, and the PAH subtype was revised to portopulmonary hypertension. Intravenous epoprostenol was temporarily initiated to stabilize the patient, and treatment was later switched to triple oral therapy with tadalafil, macitentan, and selexipag (Table 1). At the time of writing, the patient remains clinically stable.

As part of the clinical evaluation, we applied the custom gene panel HAPv.2.1 to screen the patient and her unaffected parents but found no evidence of pathogenic variants. However, WES analysis detected a novel pathogenic variant in the Tumor Necrosis Factor Receptor-Associated Factor (*TRAF2*). This gene encodes for a protein involved in the TNF-dependent activation of NF-κB (30). The variant is predicted to change cysteine to adenosine in position 417 (NM_021138.4:c.417C>A), introducing a premature stop codon and resulting in a truncated TRAF2 transcript. Analysis of the parents' exomes showed that the variant is *de novo* in the proband. This variant is also absent from the control population databases (gnomAD exomes, gnomAD genomes, Kaviar, Beacon, Bravo, ESP, 1000G phase III), and the majority of the pathogenic *in silico* tools from the dbSNFP suggested a damaging effect for this variant (Table 2).

### TNIP2 and TRAF2 Interact With Pathways Associated With PAH

**Figure 2:**
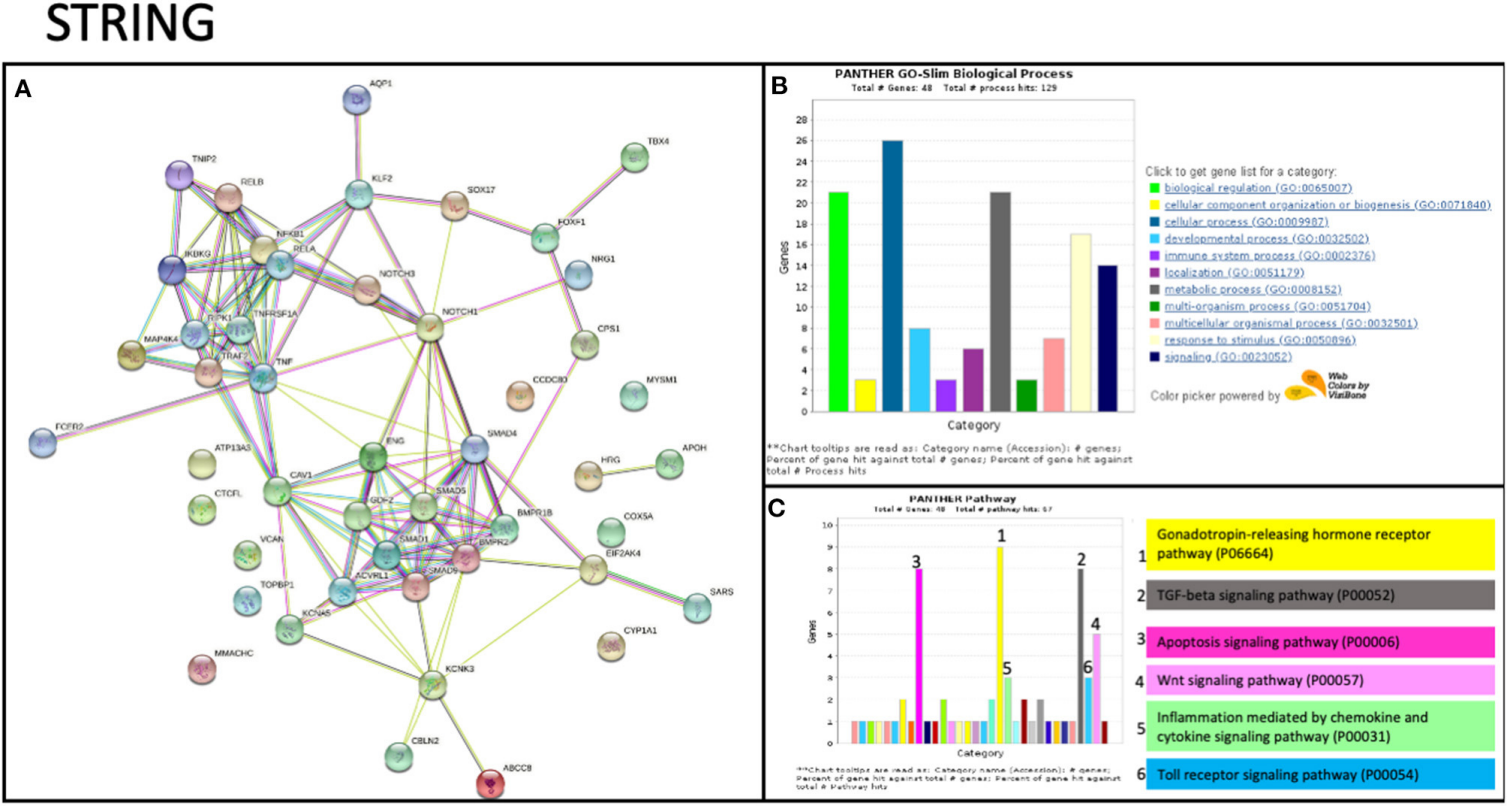
STRING analysis of TRAF2 and TNIP2 related to previously associated genes to PAH. **(A)** Protein network showing the possible interactions between all genes associated with PAH. This graph includes genes previously associated with PAH and candidate genes described in case reports/series. The strength of evidence for the relation between TRAF2 and CAV1 comes from published experimental/biochemical data and association in curated databases (see Methods). **(B,C)** Gene enrichment analysis, including biological processes **(B)** and interaction pathways **(C)** in which the STRING analysis genes are involved. Several genes are involved in the immune system process and participate in inflammation mediated by chemokine and cytokine signaling.

To date, no studies have shown evidence of *TNIP2* and *TRAF2* involvement in the pathogenesis of PAH. To determine whether these genes could potentially be interacting with components of know PAH-associated pathways, we carried out an *in silico* protein-protein screen using STRING (Figure 2A). This tool illustrates protein-protein interactions based on function and previously described associations (31). Our STRING analysis showed that TNIP2 and TRAF2 indirectly interacted with the transforming growth factor-β (TGF-β) family genes via CAV1, a known risk gene for hereditary PAH (13). Notably, BMPR2, the gene most commonly implicated in hereditary and idiopathic PAH, is part of the TGF-β family identified within the STRING cluster. As expected, we found strong associations between TNIP2 and TRAF2 with proteins involved in the NF-κB pathway (Figures 2B,C). Of the proteins associated with familial PAH, only CAV1 directly interacts with TRAF2 based on *in vitro* studies demonstrating that these proteins form a cytoplasmic protein-protein complex that modulates NF-κB activation in response to TNF (32, 33).

### Loss of TNIP2 and TRAF2 Result in Increased Pericyte Proliferation and Activation of NF-κB

TNIP2 and TRAF2 are part of a gene network responsible for regulating the NF-κB pathway's signaling activity. The NF-κB proteins exist in the cytoplasm in an inactive state and can be rapidly activated in response to harmful stimuli such as oxidative stress, bacterial lipopolysaccharides, environmental toxins, and inflammation. Once activated, the NF-κB protein complex translocates to the nucleus and binds to target genes involved in cell proliferation, survival, and the production of cytokines that regulate innate and adaptive immune responses (34). Inappropriate NF-κB activation is associated with a wide range of diseases, including cancer, autoimmune conditions such as scleroderma, and PAH (35). Given that TNIP2 and TRAF2 serve to regulate NF-κB activity, we hypothesized that the loss of these proteins would lead to NF-κB overactivation.

**Figure 3:**
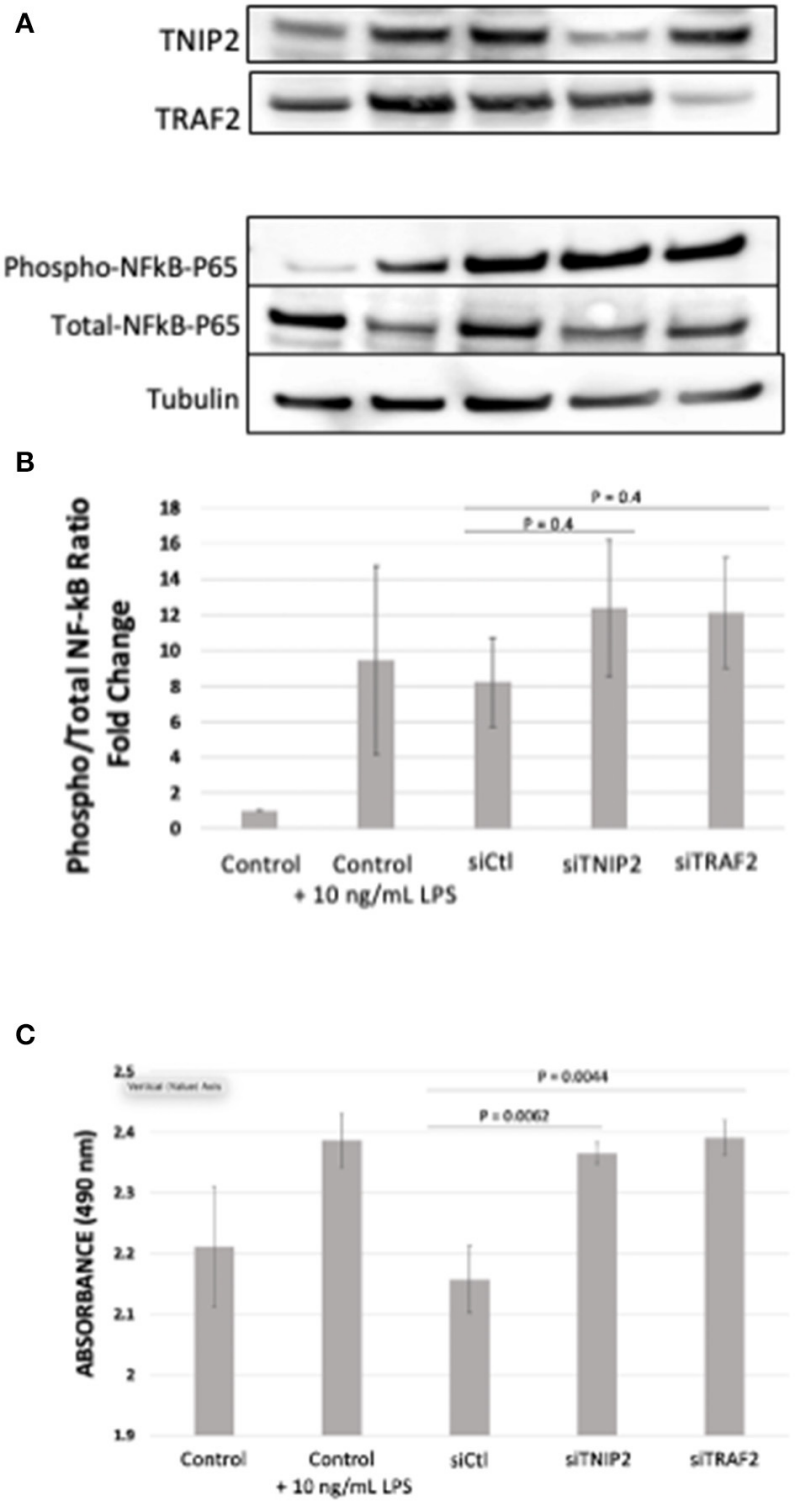
Phospho- to Total-p65-NF-κB determination and MTS Assay in lung pericytes transfected with siTNIP2 and siTRAF2. **(A,B)** Representative western immunoblots demonstrating knockdown efficiency of TNIP2 and TRAF2 in healthy lung pericytes transfected with the corresponding siRNA **(A)**, and the fold change of phosphorylated to total p65-NF-κB **(B)**. **(C)** MTS assay of healthy lung pericytes transfected with siControl (siCtl), siTNIP2, and siTRAF2 under different experimental conditions. The data in the graphs are the average of three independent experiments.

To evaluate the biological consequences of deleterious variants affecting *TNIP2* and *TRAF2*, gene silencing via siRNA transfection in human-derived pulmonary pericytes was performed. We chose to study pericytes because these cells have been shown to contribute to progressive small vessel loss and muscularization through hyperproliferation and inflammatory cytokine production (26, 27, 36). A query of our recently published pericyte RNA-seq analysis confirmed that both TNIP2 and TRAF2 are expressed in healthy and PAH pericytes, although no significant differences in expression were observed (26). Transfection of siTNIP2 and siTRAF2 resulted in >50% reduction in corresponding protein levels (Figure 3A). To determine whether TNIP2 and TRAF2 knockdown led to inappropriate activation of NF-κB, we used western immunoblotting to probe whole cell lysates for phospho-p65-NF-κB, the active form of p-65-NF-κB that becomes activated to trigger transcription of NF-κB target genes (34, 35). Stimulation with lipopolysaccharide (LPS, a known activator of NF-κB) triggered an increase in phospho-p65-NF-κB and was used as a positive control (37). Compared to baseline controls and cells transfected with a non-specific siRNA (siCtl), both siTNIP2 and siTRAF2 pericytes demonstrated a significantly increased phospho-p65-NF-κB of similar magnitude as that observed with LPS stimulation (Figure 3B). To assess the effect of TNIP2 and TRAF2 knockdown on pericyte proliferation, we used an MTS assay to quantify cell numbers under different experimental conditions over 24 h. Compared to the controls, both siTNIP2 and siTRAF2 pericytes demonstrated significantly increased cell proliferation (Figure 3C). Interestingly, the degree of proliferation observed in both siTNIP2 and siTRAF2 pericytes was similar to that of healthy pericytes stimulated with LPS.

## Discussion

**Figure 4:**
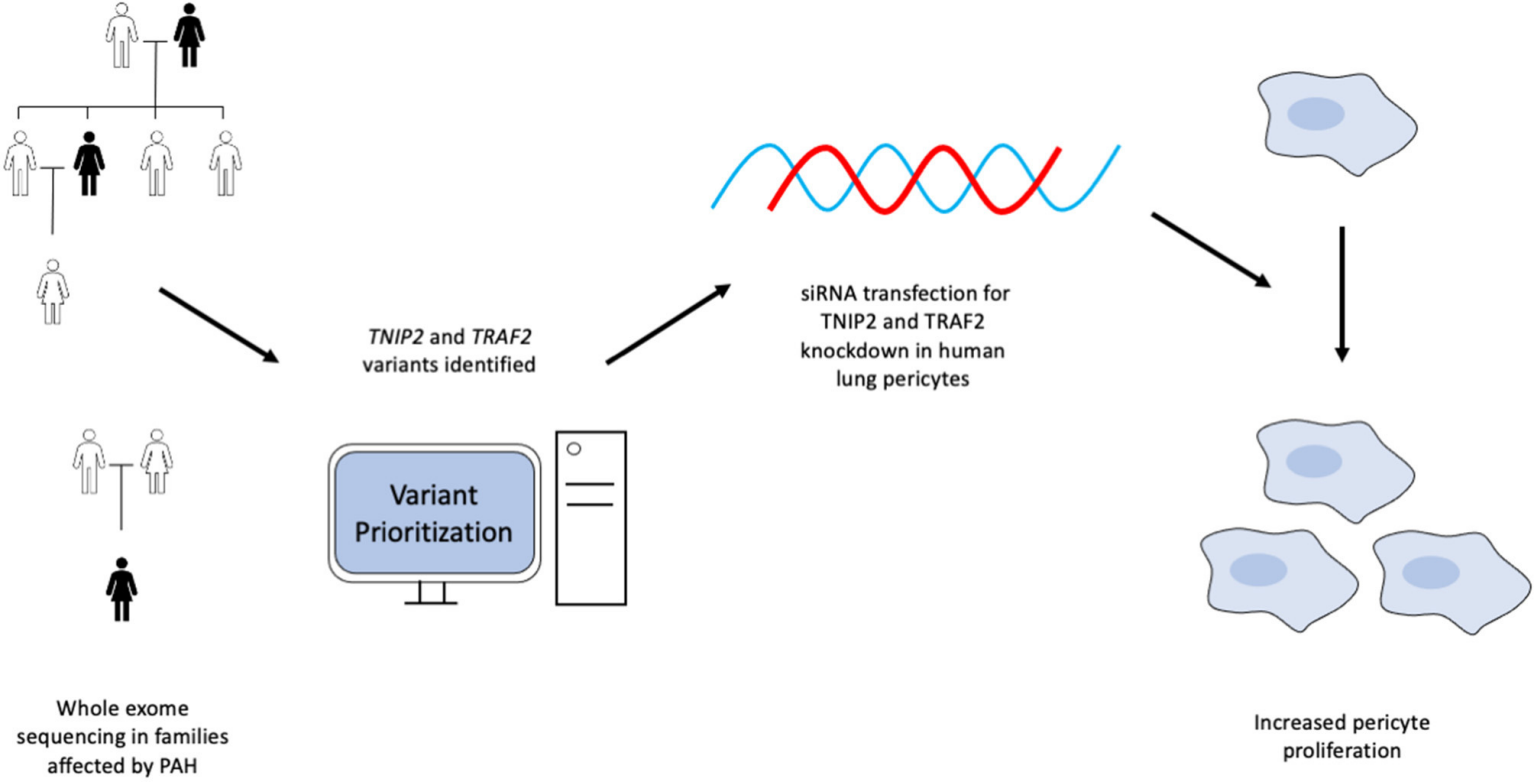
Graphical abstract summarizing the experimental design and results from the study.

In this study, we utilized a WES custom gene prioritization method optimized to identify potentially pathogenic variants in PAH patients and family members to elucidate possible genetic mechanisms influencing disease development. This approach enabled the discovery of novel variants in *TRAF2* and *TNIP2*, two genes involved in regulating the NF-κB pathway. We demonstrated that loss of TNIP2 and TRAF2 results in inappropriate NF-κB activation and increased proliferation of healthy lung pericytes through the use of siRNA-based knockdown. A graphical abstract is included in Figure 4. To our knowledge, ours is the first report to document a potential link between TNIP2/TRAF2 loss of function and PAH in humans.

Since the description of *BMPR2*, the increasing prevalence of genetic screening has shown that pathogenic variants in this gene are present in ~60% of hereditary PAH cases and 20-25% of idiopathic cases. *BMPR2*, as well as other genes (i.e., *KDR, SOX17, TBX4*), may also be involved in the development of associated forms of PAH (APAH) (38–41). Multiple groups have applied custom panels, whole-exome sequencing (WES), or even whole-genome sequencing to elucidate these mechanisms (42), which include variation in gene regulatory elements in addition to the protein-coding sequences themselves (43). Whole-genome sequencing in more extensive and more inclusive cohorts will be vital for uncovering intronic and regulatory genetic sequences that may impact PAH development in patients with unrevealing results upon initial gene panel screening. In many pulmonary hypertension centers, however, genetic analysis is not frequently offered in patients with PAH associated with other medical conditions (e.g., connective tissue diseases, congenital heart diseases), limiting the data available in this population. This practice will be essential to address future clinical guidelines to deepen our understanding of genetic variation in APAH.

As part of the clinical evaluation, our group routinely performs genetic screening for patients diagnosed with idiopathic, heritable, and associated PAH forms with suspected genetic components. We have developed a custom NGS panel of genes that is updated yearly to reflect published risk genes in PAH and recently reported our experience applying it in 300 patients, some of whom suffered from APAH (17). In all patients with inconclusive results on the gene panel, we move to WES, preferably including parents and other proband relatives, to screen for new risk genes. In recent years, multiple publications have demonstrated the role of genetic variants in the development of many PH subtypes, some of which were previously not known to have a genetic cause. For example, variants in the endoglin gene are linked with PAH associated with connective tissue disease (44), polymorphisms in estrogen-related genes may impact the risk of portopulmonary hypertension (45), and *SOX17* have been implicated in PAH associated with congenital heart disease (41). Also, variants in *CAV1, TRPM8*, and *BNP* have been linked with PH secondary to chronic obstructive pulmonary disease (46–48). These findings underscore the importance of expanding our genetic sequencing application to PH populations not traditionally thought to have a genetic predisposition.

In the present study, our WES data suggest an association between *TNIP2* and *TRAF2* variants with the development of APAH. By analyzing exome data of individuals affected with PAH compared to healthy family members, we focused on genes that correlate with phenotypic differences. After our prioritization analysis, we selected the variants in *TNIP2* and *TRAF2* based on the criteria described in Supplementary Table 1. These two variants were absent in control population databases, and the majority of the *in silico* pathogenic tools suggest a damaging effect for these variants. Both variants are highly conserved through evolution, highlighting these residues' importance in the affected protein's function. Importantly, these two genes have plausible mechanistic significance as regulators of the NF-κB pathway, previously associated with PAH (49–53). As is the case with the discovery of all novel variants, consideration of pseudocontrol human population gene frequency, conservation in orthologs through evolution, pathogenicity prediction, and biological context was necessary to establish how *TNIP2* and *TRAF2* were involved in PAH.

The family genetic analysis showed that the variant in *TNIP2* is present in both affected individuals and one unaffected member. We suspect two possible explanations for this observation. First, the variant may have incomplete penetrance or cause variable age of disease onset, allowing some carriers to be unaffected for some or all of their lives. Secondly, it may be necessary for an additional disease process to trigger the development of PAH in those who carry this variant. The high-impact nonsense *TRAF2* variant identified in a separate family was only seen in the affected proband, suggesting a *de novo* event or germinal mosaicism of the parents. Because there was no previous evidence linking pathogenic variants in *TNIP2* and *TRAF2* with PAH, we performed *in silico* analysis to search for relationships between these genes and others implicated in PAH pathogenesis. A study of the protein network by STRING showed that TRAF2 interacts with *CAV1*, a gene linked to hereditary PAH (54). In addition to STRING, there are numerous methods to identify critical gene-gene interactions and visualize pathways affected by genes of interest. Available tools include GSEA from the Broad Institute for gene list analysis and EnrichmentMap to visualize relationships between distinct pathways (55). We believe that investigators should implement these algorithms as part of ongoing genetic studies in PAH.

Inflammation has garnered increasing interest in PAH in recent years (56). Immune phenotypes characterized by differing signatures of inflammatory cytokines offer prognostic and potentially therapeutic significance (12). Antiinflammatory agents such as anakinra and tocilizumab are currently being investigated in PAH clinical trials (57, 58). NF-κB is known to regulate the expression of a wide array of inflammatory genes, and both human and animal studies indicate that NF-κB activity is increased in PAH. Core processes in PAH pathophysiology, such as aberrant pulmonary artery wall cell proliferation, apoptosis, and arterial obliteration, are attenuated with NF-κB inhibition (50). The *TNIP2* gene codes for ABIN2 (A20 binding inhibitor of NF-κB activation-2), a cytosolic protein that interacts with several membrane receptors (CD40, TNF receptor 1, and toll-like receptor 4) to suppress downstream activation of the NF-κB pathway (29, 59–61). TRAF2 belongs to a family of adaptor proteins associated with TNF receptors 1 and 2 to modulate TNF stimulation response in immune cells. Prior work supports TRAF2 as an essential mediator of cell survival and apoptosis through its capacity to activate or inhibit NF-κB, depending on the cell type (62, 63). Also, TRAF2 is involved in a membrane complex that can recruit caspases to promote apoptosis independent of the NF-κB pathway (64). Lastly, some authors have noted common hyperproliferative mechanisms driving PAH pathogenesis and cancer development (65), and pathogenic variants in TRAF proteins have been previously described in cancer (66). Specifically, *TRAF2* has suggested roles as an oncogene in epithelial cancer, osteotropic breast cancer and colon cancer (67–69), mainly through the NF-kB pathway. As evidenced by our *in vitro* studies, decreased expression of TNIP2 and TRAF2 can increase NF-κB activation in human pericytes. More importantly, pericytes demonstrated a change in proliferative activity under these conditions. These data support our hypothesis that pathogenic variants in *TNIP2* and *TRAF2* result in dysregulated NF-κB activation and could drive abnormal cell proliferation in PAH, as illustrated by the response of lung pericytes transfected with the corresponding siRNAs (Figure 3). However, a manifestation of clinically detectable PAH may require the presence of predisposing conditions (a “second-hit”), such as primary biliary cirrhosis or scleroderma, as seen in our subjects.

Our study has several limitations. First, whole-exome data was only available for a limited number of probands and their relatives, therefore limiting our power to detect novel variants potentially associated with PAH. Secondly, the technology used for our genetic analysis variants does not allow identification of variants outside of exons or exon-intron boundaries, which may hold essential regulatory elements. Additionally, our cell studies used pericytes from human donors, not from the subjects analyzed in our study. These donors do not have predisposing inflammatory or cardiac conditions, which we believe in having been essential contributors to PAH development in the probands we analyzed. However, our data support the ongoing investigation of NF-κB as a potential therapeutic target in PAH and show that our genomics-to-bench approach can be useful for expanding our view of PAH genetics.

## Conclusion

Identifying genetic biomarkers related to PAH holds the potential for a tremendous impact on patient care and the development of personalized treatments. We suggest that WES be considered for patients with associated forms of PAH that do not have causative variants identified with targeted gene panels, especially in cases with family history supportive of inherited disease. Genetic sequencing of family members with and without the disease, when feasible, will provide additional power to link genotype, phenotype, and endotype in PAH. Understanding the genetic underpinnings that predispose individuals to immune dysregulation in the pulmonary vascular wall will be paramount for advancing targeted therapies for this deadly disease. Variants in *TRAF2* and *TNIP2* may contribute to hyperproliferation and increased NF-κB activation in some individuals with PAH, however further studies are needed to describe the possible relationship of these genes with aberrant immune activity.

## Data Availability Statement

The datasets presented in this study can be found in online repositories. The names of the repository/repositories and accession number(s) can be found below: NCBI BioProject, accession no: PRJNA702898 (ACCESSION PRIVATE).

## Ethics Statement

All patients involved in this study gave their informed consent to participate. Samples were obtained from the Spanish PAH registries (REHAP and REHIPED), and the Ethical Committee approved the project of scientific research of the Hospital Universitario La Paz, Madrid (PI-1210, PI-3996).

## Author Contributions

JT and VDJP designed and supervised the work. NG, PA, and JT performed all genetic studied and NGS analysis. SP, DC, SA, HP, and AC performed all the functional and *in vitro* studies. ACU, NO, PE, and PL reviewed all the clinical features of the patients. JN performed the microarray analysis. JT, VDJP, SP, NG, and DC wrote the manuscript. All authors contributed to the article and approved the submitted version.

## Conflict of Interest

The authors declare that the research was conducted in the absence of any commercial or financial relationships that could be construed as a potential conflict of interest.
