# Supplementary figures and images for: Novel TNIP2 and TRAF2 Variants Are Implicated in the Pathogenesis of Pulmonary Arterial Hypertension
Source: Front Med (Lausanne). 2021 Apr 30;8:625763. doi: 10.3389/fmed.2021.625763 (PMC8119639; doi:10.3389/fmed.2021.625763)

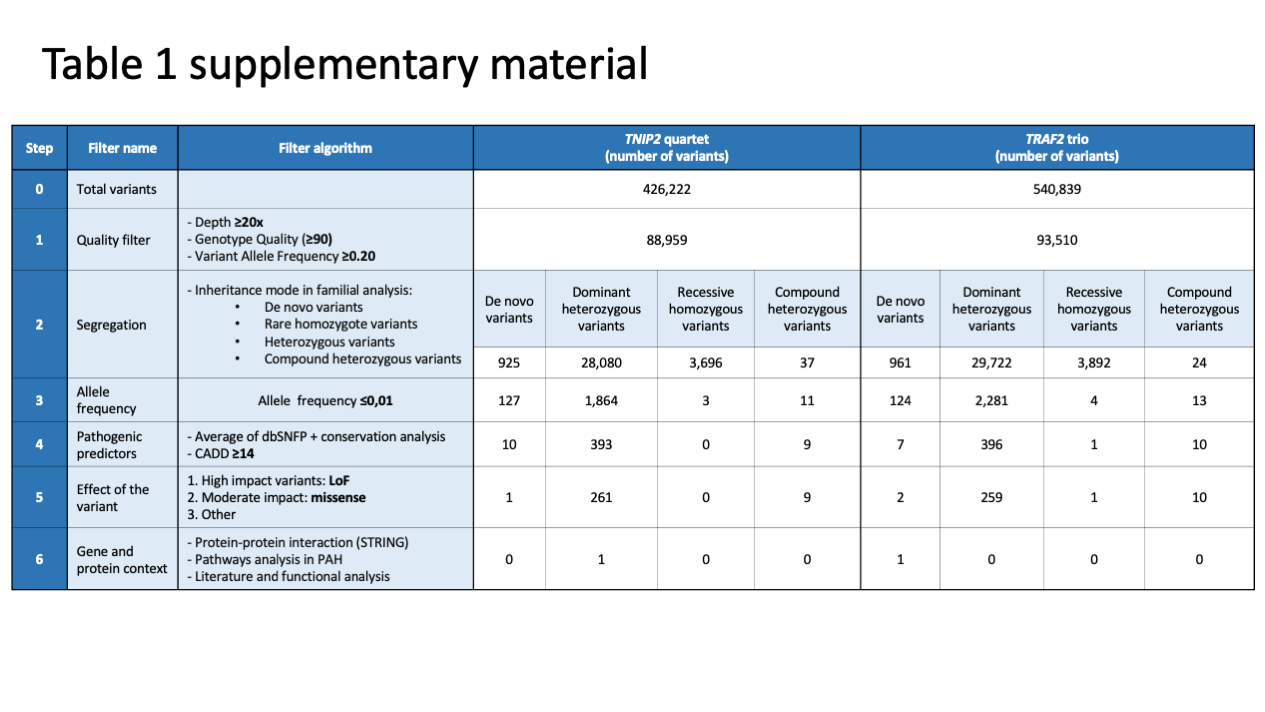

Supplement: Supplementary file 1 [file Image_1.tiff]
